# Does Perceived Physical Attractiveness in Adolescence Predict Better Socioeconomic Position in Adulthood? Evidence from 20 Years of Follow Up in a Population Cohort Study

**DOI:** 10.1371/journal.pone.0063975

**Published:** 2013-05-22

**Authors:** Michaela Benzeval, Michael J. Green, Sally Macintyre

**Affiliations:** Social and Public Health Sciences Unit, Medical Research Council, Glasgow, United Kingdom; Federal University of Rio de Janeiro, Brazil

## Abstract

There is believed to be a ‘beauty premium’ in key life outcomes: it is thought that people perceived to be more physically attractive have better educational outcomes, higher-status jobs, higher wages, and are more likely to marry. Evidence for these beliefs, however, is generally based on photographs in hypothetical experiments or studies of very specific population subgroups (such as college students). The extent to which physical attractiveness might have a lasting effect on such outcomes in ‘real life’ situations across the whole population is less well known. Using longitudinal data from a general population cohort of people in the West of Scotland, this paper investigated the association between physical attractiveness at age 15 and key socioeconomic outcomes approximately 20 years later. People assessed as more physically attractive at age 15 had higher socioeconomic positions at age 36– in terms of their employment status, housing tenure and income - and they were more likely to be married; even after adjusting for parental socioeconomic background, their own intelligence, health and self esteem, education and other adult socioeconomic outcomes. For education the association was significant for women but not for men. Understanding why attractiveness is strongly associated with long-term socioeconomic outcomes, after such extensive confounders have been considered, is important.

## Introduction

Numerous studies have indicated that individuals who are perceived to be more physically attractive may be more successful in a number of life domains. For example, studies have shown a ‘beauty premium’ in relation to educational outcomes [1], gaining employment [2], [3], occupational success [4], [5], and income [6], [7], [8]. It has also been shown that attractiveness is important in choice of partner [4], [9], and that more attractive women are more likely to marry [10], [11] and to marry partners of a higher social status, although for men the evidence is more mixed [11], [12]. As well as having more positive socioeconomic outcomes, people regarded as attractive are believed to have other positive attributes. For example, compared to those rated as unattractive, those more attractive are also perceived to have more positive character traits [13], [14], to have greater self esteem and self confidence [4] and to have higher IQ [15], [16], [17].

However, much of the evidence in this field is experimental [5], [15], [18]. Some studies are based on ‘hypothetical’ experiments i.e. participants are shown photographs of people and asked to decide whether to employ them or give them a pay rise [2], [3]. Other studies are based on very select groups of people, for example, school photographs are rated for the students’ attractiveness and then alumni’s subsequent employment and other outcomes analysed [11], [19], [20]. However, there is some evidence to suggest that such experiments yield stronger effects of attractiveness on outcomes than correlation studies based on observations of ‘real life’ situations [6]. A small number of general population studies have been conducted. For example, a strong association was found between (interviewer rated) attractiveness and income, education, employment and occupational prestige in a cross-sectional study in the USA [1], while a longitudinal Canadian study found an association between attractiveness and income for men but not for women [21] and attractiveness was associated with lower labour force participation in a very long term follow up of women in California [22]. A key issue in observational studies such as these is whether an appropriate range of confounders are considered. For example, in one of the most comprehensive longitudinal general population studies to date, Harper found that when academic ability and sociability were controlled for, the association between attractiveness and pay became insignificant, although other associations remained positive [10].

In a cross-sectional investigation we found that attractiveness in a cohort of 1,500 adolescents aged 15 was associated with having a more affluent family background [23]. Understanding what role, if any, attractiveness might play, in the complex pathways between childhood and adulthood socioeconomic circumstances, is important. In this paper therefore we examine the socioeconomic outcomes for these adolescents, 20 years later, to identify the long-term correlates of attractiveness in ‘real life’ situations. In particular we investigated the association between attractiveness at age 15 and education, employment status, occupation, marital status and partner’s social status, housing tenure (as a proxy for wealth) and household income at age 36. For all of these associations we controlled for key confounders, namely parental socioeconomic background, and own self esteem, health and IQ. Given that some studies have found different associations between attractiveness and socioeconomic circumstances for men and women [10], while others have not [4], we also investigated whether the findings varied by gender.

## Materials and Methods

### Study Sample

The data employed in this analysis were from the youth cohort of the Twenty-07 Study [24], which included 1,515 people born around 1972 with a mean age of 15.7 years at the baseline interviews in 1987/88. It has two subsamples: the regional sample, a two-stage stratified random sample of people living in and around the city of Glasgow, and the localities sample of people from two specific areas of Glasgow. Baseline respondents have been shown to be representative of the general population of the sampled area [25]. This paper focuses on data from the baseline interviews and the fifth follow-up visit in 2007/8 when the mean age of respondents was 36.7 years. By the fifth wave, 30 respondents had died and 942 took part; 63 percent of those still alive [26]. Respondents who remained in the study were more likely to be female, to have lived in more affluent households at baseline and to have better health than those who dropped out of the study [26].

### Ethics Statement

Ethical approval for the overall study and baseline data collection was granted in 1986 by both the GP Sub-Committee of the Greater Glasgow Health Board Area Medical Committee and the Ethical Sub-Committee of the West of Scotland Medical Committee. Ethical approval for the fifth wave of fieldwork was given by Tayside Committee on Medical Research Ethics A. At Wave 1, since the respondents were 15 or 16 years old, informed written consent was sought from their parents as well as the respondents themselves. At the fifth wave informed written consent was given by the respondents.

### Measures

#### Attractiveness at age 15

Attractiveness was rated at baseline on a scale of 1 (very attractive) to 7 (very unattractive). The instructions to the interviewers read: ‘Look at the respondent and ask yourself how good looking is he/she? There is a natural tendency in us all to want to rate people more rather than less attractive. Do not feel bad about rating someone less than average in the ‘good looks’ stakes; he or she might have a great personality’. This measure of attractiveness therefore was based on the overall appearance of the respondent rather than specific aspects – such as their face, body shape, height etc.

Each respondent had two home visits and parents were also interviewed, which meant that up to three interviewers visited the home. All three interviewers (one nurse and two social interviewers) were asked to assess the respondent’s attractiveness if they saw him/her (respondents were not necessarily present for their parents’ interview). Our measure therefore is based on a real-life encounter rather than on photographs. This makes it a much more naturalistic assessment, but may mean interviewers’ perceptions were influenced by the home environment. We discuss this issue further below. The mean of the scores across all three interviewers was employed in these analyses and has been shown to correlate well with each individual rating [23]. In this paper the scores have been reversed so that a higher score represents greater attractiveness.

#### Adult Socioeconomic Position (SEP) at age 36

During the 2007/8 interviews respondents were asked a range of questions regarding their own current SEP. We examined six key adult outcomes. Educational attainment was based on whether or not the respondent stayed in school beyond the UK school leaving age of 16 years. Employment status was coded into three categories as full-time, part-time, or not employed (which included caring for home and family, full-time education, and being unemployed or out of work due to ill health). Occupation-based social class was coded according to the 1980 UK Registrar General’s classification [27], for the respondent’s own current or most recent job, and dichotomised into manual (III manual, IV & V) and non-manual (I, II & III non-manual) categories. Given debates in the literature about whether attractive people are not only more likely to marry but more likely to marry someone of higher social status, we combined respondents’ partnership status and their partner’s current or most recent occupation. This resulted in a single variable that distinguished between those who were single (including divorced, widowed and separated), married/co-habiting with someone in a manual occupation, or married/co-habiting with someone in a non-manual occupation. Housing tenure was dichotomised to distinguish between home-owners and those in rented or other types of accommodation. Weekly household income after tax was directly reported by respondents, equivalised to take account of household composition [28] and split into tertiles.

#### Confounders

In order to consider the longitudinal association between attractiveness and adult SEP, it was important to take account of key confounders, including socioeconomic circumstances and health in childhood, IQ, and self esteem. All of these factors have been shown to be correlated with both attractiveness and adult SEP. Some studies also consider height as a confounder. However, since height is a key component of attractiveness, and we wished to investigate the association between a person’s whole appearance and these outcomes, we have not included height.

A parental interview was conducted in 1987/88 from which the parental SEP measures were derived. Answers were coded in the same way as the indicators of respondents’ SEP with the following exceptions. The marital status variable simply differentiated between respondents who had a single parent or guardian and those whose parents or guardians were married or co-habiting. Parental education and household occupational class were based on the parent with the higher status in couple households. Data on parental income were collected by a banded income question. Parents reported whether their weekly household income after tax was less than £50, £50–99, £100–149, £150–199, £200–249, £250–299, £300–349, £350–399, £400–449, £450–499 or greater than £500. The mid-point of the chosen band was equivalised for household composition [28] and then split into tertiles.

Self-esteem at age 15 was measured with a self-report scale based on Rosenberg's inventory [29] by asking respondents to assess agreement on a 5-point scale with 10 items such as ‘I like myself’ or ‘I am able to do things well’. It did not include items relating to body image. Responses were summed to create a self-esteem score ranging from 10 to 50, with higher scores representing higher self-esteem [30]. We included two measures of overall health status at age 15. First, respondents were asked to assess their own health as ‘good’, ‘fairly good’ or ‘not good’ and secondly, they were asked whether or not they had any sort of limiting longstanding illness.

IQ was measured at age 36 using part I of the Alice Heim 4 test of general intelligence (AH4). AH4 has been used widely in cohort studies in the UK as a reliable and valid measure of general cognitive ability. The test is based on 65 items, including verbal and numerical reasoning, of which the participant completes as many items as possible in ten minutes. Administration and scoring were carried out according to instructions in the test manual [31]. Unfortunately, we did not have a measure of general IQ when respondents were 15. Our measure at age 36 will have been influenced by education and SEP in childhood. However, since we are trying to assess the association between attractiveness and adult SEP independent of education, background SEP and IQ, we believe that this is an adequate measure for our purposes.

### Data Analysis

Analyses were performed in Mplus 7 [32] and were either multinomial or binary logistic regressions, depending on the number of categories in the outcome variable. All models were adjusted for gender and an interaction between gender and attractiveness was included in all models for that outcome if it was found to be significant at the p<0.05 level. Once an interaction was found at this level it was retained in subsequent models until it was no longer significant at the p<0.1 level.

Complex standard errors were used to adjust for sample stratification. In order to correct for bias due to drop-out respondents participating in the 2007/8 interviews were weighted to the living baseline sample using inverse probability weighting [33]. Item missingness was relatively low for each variable employed; the highest levels of missingness – between 5 and 6% - were for parental and own household income and for the IQ score at age 36. Overall, however, 27.7% of respondents had at least one missing item and hence item-missing values were multiply imputed in addition to the weighting. An unrestricted model [34] of all the analysis variables (including the weighting variable), and some additional, mainly health, auxiliary variables, was used to create 25 imputed data-sets. Analysis results were averaged across these data-sets [35]. This imputation method allows each variable to contribute to the prediction of every other variable and adjusts for non-random differences in the missing compared to the observed values so long as these differences can be predicted by the other variables in the model.

In order to examine the attenuation of the attractiveness associations with different sets of explanatory factors, these were introduced into the model in stages. Initially, sex-adjusted univariate associations between attractiveness at age 15 and each SEP outcome variable were examined (Model 1). At stage 2, parental SEP measures were added to these models (Model 2). Next, education was controlled for (except where education was the outcome; Model 3), to see whether it might mediate the associations between attractiveness and other adult outcomes. After this, adult occupational class and marital status were also entered into the models (except where they were the outcomes; Model 4) to see how much of the attractiveness advantage was mediated by other aspects of adult SEP. Finally, self esteem, self-assessed health, limiting longstanding illness and general intelligence were added to the models to control for their potential confounding effect (Model 5).

Associations with attractiveness are primarily presented as the odds ratios (OR) and 95% confidence intervals associated with a 1-point higher attractiveness rating. For the two continuous variables – IQ and self esteem - in Table 1 the beta coefficients are presented instead, in standard deviation units (i.e. the number of standard deviation units change in that variable associated with a 1-point higher attractiveness rating). Values for gender interactions represent the additional effect of attractiveness for females, over and above the main effect of attractiveness.

**Table 1:** Population proportions, means and sex-adjusted associations of main confounders and attractiveness at age 15.

	PopulationCharacteristics^a^		Sex-Adjusted Association with 1 unitincrease in Attractiveness rating at age 15		
	N/Mean	%/S.D	OR/Beta	95% CI/S.E.	P-Value
**Attractiveness Rating** b	4.7	0.8	−	−	
**Female** (ref: male)	487	51.7	1.43	1.21–1.69	<0.001
**Parental Employment** (ref: Full-time)					
Part-time	81	8.6	0.78	0.61–1.06	0.053
Not employed	207	22.0	0.54	0.38–0.77	0.001
**Parental Manual Occupational Class** (ref: Non-Manual)	367	39.0	0.69	0.54–0.88	0.003
**Parental Marital Status:** single (ref: Married/Cohabiting)	145	15.4	0.78	0.62–0.98	0.035
**Parental Education:** Left at 16 or earlier (ref: post-16)	601	63.8	0.66	0.53–0.80	<0.001
**Parental Tenure:** Rent/other (ref: owner/mortgage)	532	56.5	0.54	0.44–0.67	<0.001
**Parental household Income** (ref: top tertile)					
Middle tertile	325	34.5	0.73	0.57–0.95	0.018
Bottom tertile	311	33.0	0.50	0.36–0.69	<0.001
**Self-esteem age 15** b	36.0	5.1	0.03	0.05	0.572
**Own IQ score (AH4) at age 36** b	38.1	10.2	0.27	0.06	<0.001
**Self-Assessed Health:** not good (ref: good/fairly good)	34	3.6	0.34	0.16–0.74	0.007
**Limiting Longstanding Illness:** Yes (ref: no)	99	10.5	0.73	0.51–1.03	0.076

a The means and population proportions in this column are weighted values, averaged across the 25 imputed data-sets (total n = 942).

b As these variables were continuous rather than categorical, results are presented as means and beta coefficients in standard deviation units, rather than as proportions and odds ratios.

## Results

Table 1 displays population proportions, means, and sex-adjusted associations with attractiveness for all confounders from the weighted, imputed, data. The average attractiveness score was 4.7 (s.d. 0.8) out of a possible total of 7. Girls were more likely to be rated as attractive at age 15 than boys. All of the indicators of parental SEP at age 15 were associated with attractiveness ratings at that age. Respondents who were rated more attractive tended to live in more favourable socioeconomic circumstances during their childhood. There was a statistically significant association between adult IQ and attractiveness, such that those who were perceived as more attractive at age 15 had higher IQ scores at age 36 (p<0.001). With respect to health at age 15 more attractive respondents were less likely to rate their health as ‘not good’ (p = 0.007), and there was a similar, though non-significant, trend for having a limiting longstanding illness (p = 0.076). However, there was no association between attractiveness and self esteem at age 15 (p = 0.454).

Table 2 shows the associations between ratings of attractiveness at age 15 and own adult socioeconomic outcomes, whilst Table 3 shows the association with household adult SEP outcomes. Model 1 shows that there was a statistically significant association between attractiveness and most of the SEP outcomes, with respondents who were rated as more attractive at age 15 being in more favourable socioeconomic circumstances at age 36. For example, a one unit increase in attractiveness was associated with an OR of 0.45 (P<0.001) for being in the bottom, compared to the top, income tertile at age 36. There were interactions between gender and attractiveness for two socioeconomic outcomes – education (P = 0.029) and occupational class (p = 0.025) – such that attractive females had stronger occupational advantages than attractive males and had an advantage in education where attractive males did not.

**Table 2:** Adjusted relationships between 1 unit increase in youth attractiveness rating and own adult SEP outcomes.

SEP at age 36	Model 1^a^			Model 2^b^			Model 3^c^			Model 4^d^			Model 5^e^		
	OR	95% CI	P-Value	OR	95% CI	P-Value	OR	95% CI	P-Value	OR	95% CI	P-Value	OR	95% CI	P-Value
**Education** (ref: post-16)															
Left at age 16or earlier	0.81	0.63–1.04	0.095	0.96	0.74–1.25	0.776	−	−	−	1.11	0.85–1.44	0.459	1.24	0.93–1.65	0.148
Gender Interaction(ref: Male)	0.60	0.37–0.95	0.029	0.62	0.39–0.98	0.039	−	−	−	0.66	0.41–1.06	0.084	0.61	0.38–0.99	0.044
**Employment** (ref: Full-time)															
Part-time	0.74	0.58–0.94	0.014	0.74	0.60–0.91	0.004	0.75	0.61–0.92	0.005	0.79	0.64–0.97	0.028	0.80	0.65–0.99	0.039
Not employed	0.46	0.31–0.67	<0.001	0.51	0.36–0.73	<0.001	0.51	0.35–0.72	<0.001	0.62	0.45–0.87	0.005	0.66	0.48–0.91	0.012
**Own Occupational Class** (ref: Non-Manual)															
Manual	0.58	0.44–0.76	<0.001	0.64	0.49–0.85	0.002	0.55	0.44–0.70	<0.001	0.57	0.45–0.73	<0.001	0.60	0.47–0.79	<0.001
Gender Interaction(ref: Male)	0.58	0.36–0.93	0.025	0.62	0.38–1.01	0.053	n/s	−	−	n/s	−	−	n/s	−	−

a Model 1 Adjusted for gender.

b Model 2 additionally adjusted for all indicators of parental SEP.

c Model 3 additionally adjusted for own education (except where education is the outcome).

d Model 4 additionally adjusted for own adult occupational class (except where own class is the outcome), and adult marital status.

e Model 5 additionally adjusted for adult IQ, self-assessed health, limiting longstanding illness, and self-esteem.

**Table 3:** Adjusted relationships between 1 unit increase in youth attractiveness rating and household adult SEP outcomes.

SEP at age 36	Model 1^a^			Model 2^b^			Model 3^c^			Model 4^d^			Model 5^e^		
	OR	95% CI	P-Value	OR	95% CI	P-Value	OR	95% CI	P-Value	OR	95% CI	P-Value	OR	95% CI	P-Value
**Marital Status** (ref: Partner of Non-Manual Class)															
Partner ofManual Class	0.56	0.44–0.73	<0.001	0.66	0.51–0.85	<0.001	0.66	0.51–0.85	0.001	0.66	0.51–0.84	<0.001	0.63	0.49–0.82	0.001
Single	0.56	0.42–0.76	<0.001	0.58	0.44–0.77	<0.001	0.58	0.44–0.77	<0.001	0.61	0.46–0.81	<0.001	0.65	0.49–0.84	0.001
**Tenure** (ref: owner/mortgage)															
Rent/other	0.47	0.32–0.70	<0.001	0.55	0.38–0.78	0.001	0.56	0.39–0.81	0.002	0.69	0.47–1.00	0.049	0.70	0.48–1.01	0.055
**Income** (ref: top tertile)															
Middle tertile	0.84	0.69–1.02	0.075	0.90	0.73–1.12	0.364	0.91	0.73–1.13	0.386	0.99	0.78–1.27	0.943	1.01	0.80–1.28	0.946
Bottom tertile	0.45	0.33–0.62	<0.001	0.56	0.42–0.76	<0.001	0.58	0.43–0.77	<0.001	0.74	0.54–1.03	0.071	0.77	0.55–1.07	0.125

a Model 1 Adjusted for gender.

b Model 2 additionally adjusted for all indicators of parental SEP.

c Model 3 additionally adjusted for own education.

d Model 4 additionally adjusted for own adult occupational class, and adult marital status (except where marital status is the outcome).

e Model 5 additionally adjusted for adult IQ, self-assessed health, limiting longstanding illness, and self esteem.

Adjusting for indicators of parental SEP at age 15 (Model 2) slightly attenuated these associations without substantially changing any of them, though the interaction between gender and attractiveness for occupational class did go just beyond traditional significance levels (p = 0.053). When the respondents’ own education was adjusted for (Model 3) the associations between attractiveness ratings and the other socioeconomic outcomes were again largely unchanged, suggesting that education is not an important mediator in the association. The only exception was again for the interaction between gender and occupational class, which became insignificant once education was taken into account suggesting that this interaction occurred because educational achievement was also more strongly associated with attractiveness for women than for men. With adjustment for own occupational class and marital status (Model 4) the associations between attractiveness ratings and housing tenure, income and employment status at age 36 were all weakened and that for income passed traditional significance levels (p = 0.071). When adult IQ, baseline health and self-esteem were taken into account the association with housing tenure also passed traditional significance levels (p = 0.055), but the other associations remained largely unchanged.

**Figure 1:**
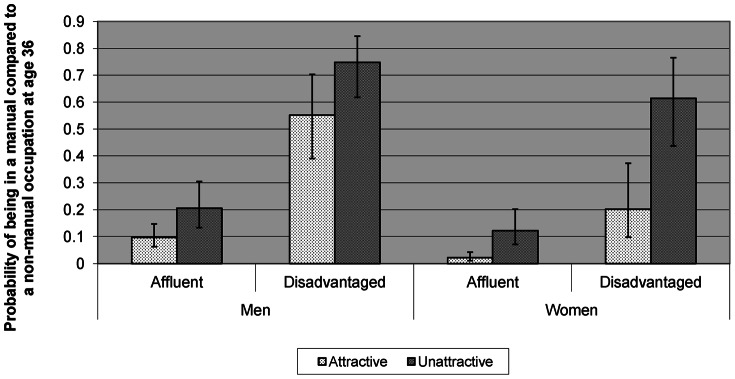
Probability of being in a manual, as opposed to non-manual class, at age 36 by attractiveness rating.

By way of illustration Figure 1 shows the estimated probabilities (from Model 2) of being in a manual, as opposed to non-manual, occupation at age 36 for respondents with specific characteristics. Estimates labelled ‘attractive’ are for respondents with attractiveness ratings 1 point above the mean (4.7), and those labelled ‘unattractive’ are for those with ratings 1 point below the mean. Estimates labelled ‘affluent’ are for respondents whose parents were categorised as full-time employed, in a non-manual social class, married or co-habiting, having post-16 education, owning their home and in the top income tertile. Those labelled ‘disadvantaged’ are for respondents whose parents were unemployed, from a manual social class, single, had not received post-16 education, did not own their homes and were in the lowest income tertile. For respondents from ‘affluent’ backgrounds where the overall odds of being in a manual, as opposed to non-manual, class at age 36 are low, the effect of attractiveness is relatively low, whereas for those from ‘disadvantaged’ households attractiveness can be seen to have a considerable effect on the probability of being in a manual occupational class at age 36, over and above the effect of background SEP. This was especially true for women who appeared unlikely to be in a manual class at age 36 unless they were both unattractive and came from a disadvantaged background.

## Discussion

This analysis builds on previous cross-sectional analyses of these data at age 15, which demonstrated that children from higher SEP families were rated as more attractive by up to three independent interviewers [23]. This longitudinal analysis of the same people 20 years later showed that, over and above this socioeconomic advantage, people who were considered attractive as teenagers had higher socioeconomic positions as adults. The more attractive a child was rated at age 15, the higher their socioeconomic position at age 36. One exception was that attractiveness was only associated with staying in education for women and not for men. Adding other adult SEP variables did attenuate these associations modestly, but including potential key confounders such as IQ, baseline health and self-esteem resulted in little further attenuation.

In general, these findings are consistent with other studies. For example, in a meta-analytic review of a range of behaviours and outcomes, Langlois and colleagues [4] found that adults rated as attractive had more occupational and dating success, and were slightly more intelligent, than those rated as less attractive. However, many of these studies were small, did not include adults beyond college age and were not based on longitudinal data from general populations. There are, however, a few large scale longitudinal studies that have investigated the advantages of being perceived as attractive for different outcomes. Fletcher [36] found a significant association between interviewer-rated attractiveness and average earnings at age 22 for 4,000 people in the USA who completed their education at high school in the National Longitudinal Study of Adolescent Health. In the National Child Development Study, a UK study of nearly 8,000 respondents, Harper found a significant association between teacher assessments of attractiveness at ages 7 and 11 years and earnings and marriage outcomes at age 33 years [10]. There were gender differences with unattractive men experiencing a greater ‘pay penalty’ compared to attractive men than unattractive women did compared with attractive women. However, the opposite was true with marriage, for which women experienced greater effects. The associations with earnings were insignificant once academic ability was accounted for in the modelling. Harper [10], and a few US studies based on the National Longitudinal Study of Youth and the Panel Study of Income Dynamics, have also examined the role of obesity [37], [38], [39] and height [40], as proxies for attractiveness, on earnings. Overall, these studies showed similar effects, with taller or slimmer respondents having better wages and being more likely to be married than shorter or obese individuals.

Our finding of a lack of association between attractiveness and self esteem is perhaps counterintuitive. However, the literature also finds inconsistent results, with some studies finding positive associations [4], others only finding associations for women but not for men [41], while others find no association [42] or even negative ones [43].

This analysis adds to the previous literature by investigating the association between attractiveness and a wide range of adult socioeconomic outcomes after a long follow-up period of 20 years. It is based on a representative sample of nearly 1,000 teenagers, and we were able to control for a wide range of confounders, in particular the socioeconomic circumstances of the respondent’s family during childhood, their general intelligence, baseline health and self esteem (although the latter was insignificant).

There are, however, a number of limitations with this analysis. Attractiveness was rated by up to three interviewers and since no photographs were taken and no other independent verification possible, it is difficult to know how valid the ratings were. However, taking the average rating of three different interviewers for each respondent reduces the impact of differential ratings by individual observers. The interviewers all rated the respondents’ attractiveness in their own homes, so there is a possibility that their assessments were influenced by the socioeconomic surroundings. However, adjustments were made for the baseline socioeconomic context using a wide range of measures, which should control for this possibility. Moreover, this ‘real-world’ rating of attractiveness is likely to reflect actual assessments in ‘real-world’ social interactions more effectively than assessing photographs which is the main approach in much of this literature. Our measure of attractiveness is also likely to reflect an overall assessment of the respondent’s appearance, given the interviewer instructions, rather than focusing on one specific aspect – such as facial features, height or body shape – as many previous studies have done.

By the fifth wave of the study when the socioeconomic outcomes were measured, 2% of study participants had died and 36% had dropped out. Those who dropped out were less affluent (and attractive) at age 15 than those who remained in the study for the full 20 years. To correct for possible bias due to drop out, inverse probability weights were employed to weight the analysis sample back to the baseline sample. There was also some item missingness, which was addressed using multiple imputation. Sensitivity analyses showed that the results presented here were similar to those for complete cases. Both of these approaches to address missingness assume data are missing at random, i.e. that non-random missingness can be predicted by other variables in the models. Given the wide range of variables employed in this analysis and other, particularly health variables, included in the imputation models, we believe this is a fair assumption.

The posited pathways between attractiveness and subsequent socioeconomic outcomes fall into three main explanations. First, gatekeepers to key socioeconomic opportunities may be influenced by the attractiveness of individuals. This is because cultural norms, stereotypes and expectations about attractiveness are likely to influence both the judgement and the treatment of attractive versus unattractive individuals [4]. For examples, teachers may provide more help and support to attractive students, perhaps because they judge them to have greater intelligence or academic competence [4]. In the world of paid work, employers interviewing candidates for a position or discussing wages may look more favourably on attractive candidates, either because they perceive them to have more positive attributes or because they believe customers may do so. There is some evidence to support this pathway. For example, analyses of occupational earnings suggests that attractiveness does play a greater role in the wages of those in customer-orientated industries than in other kinds of occupations [10]. Secondly, perceived attractiveness may lead to individuals having a greater sense of self worth and self esteem which in turn enhances their success in education, job and marriage markets. Evidence from meta-analyses suggests that attractive adults are more extroverted, have better social skills and higher self esteem and self confidence than those rated as less attractive [4]. However, we did not find an association at age 15 between self esteem and attractiveness, which suggests that these characteristics may not be a key mechanism or that our measure of self esteem in adolescence was inadequate and/or that a self esteem advantage has not developed at age 15. Finally, attractiveness may be correlated with other key determinants of adult SEP outcomes, in particular, intelligence [16], [17] and health [44], [45], and therefore may not be a direct cause of the association but a confounder. Moreover, theories of mate selection suggest that attractiveness, health and intelligence may ‘coevolve’ because of assortative mating. This theory suggests that attractive women tend to choose intelligent men because of their ability to acquire resources (and vice versa), and their children inherit both characteristics [4], [16], [17]. Similarly, it has been suggested that healthy men may choose attractive partners, which again may pass both characteristics on to children [46]. However, in our study, while attractiveness, health and IQ were correlated, health and IQ did not attenuate the association with adult SEP after adjusting for parental SEP and education, suggesting that this may not be an important pathway. The evidence presented here suggests that the most likely pathway between attractiveness and health is the role of gatekeepers. Further research to examine this potential pathway in more detail is required to improve understandings of the mechanisms that drive the strong associations observed here.

### Conclusion

This paper has shown that perceived attractiveness at age 15 is significantly associated with a wide range of adult socioeconomic outcomes even after controlling for the fact that such teenagers tended to have a better start in life. It adds weight to existing evidence from hypothetical experiments, specific small scale studies and a few general population studies, that adolescent attractiveness assessed in ‘real life’ situations can have long term associations with key outcomes for adults. Investigating the pathways that might mediate this association, in particular the role of gatekeepers, is required.
